# Integrated Machine Learning Decision Tree Model for Risk Evaluation in Patients with Non-Valvular Atrial Fibrillation When Taking Different Doses of Dabigatran

**DOI:** 10.3390/ijerph20032359

**Published:** 2023-01-29

**Authors:** Yung-Chuan Huang, Yu-Chen Cheng, Mao-Jhen Jhou, Mingchih Chen, Chi-Jie Lu

**Affiliations:** 1 Graduate Institute of Business Administration, Fu Jen Catholic University, New Taipei City 242062, Taiwan; 2 Department of Neurology, Fu Jen Catholic University Hospital, Fu Jen Catholic University, New Taipei City 24352, Taiwan; 3 Artificial Intelligence Development Center, Fu Jen Catholic University, New Taipei City 242062, Taiwan; 4 Department of Information Management, Fu Jen Catholic University, New Taipei City 242062, Taiwan

**Keywords:** cardioembolic stroke, arrhythmia, anticoagulant agents, machine learning, decision tree

## Abstract

The new generation of nonvitamin K antagonists are broadly applied for stroke prevention due to their notable efficacy and safety. Our study aimed to develop a suggestive utilization of dabigatran through an integrated machine learning (ML) decision-tree model. Participants taking different doses of dabigatran in the Randomized Evaluation of Long-Term Anticoagulant Therapy trial were included in our analysis and defined as the 110 mg and 150 mg groups. The proposed scheme integrated ML methods, namely naive Bayes, random forest (RF), classification and regression tree (CART), and extreme gradient boosting (XGBoost), which were used to identify the essential variables for predicting vascular events in the 110 mg group and bleeding in the 150 mg group. RF (0.764 for 110 mg; 0.747 for 150 mg) and XGBoost (0.708 for 110 mg; 0.761 for 150 mg) had better area under the receiver operating characteristic curve (AUC) values than logistic regression (benchmark model; 0.683 for 110 mg; 0.739 for 150 mg). We then selected the top ten important variables as internal nodes of the CART decision tree. The two best CART models with ten important variables output tree-shaped rules for predicting vascular events in the 110 mg group and bleeding in the 150 mg group. Our model can be used to provide more visualized and interpretable suggestive rules to clinicians managing NVAF patients who are taking dabigatran.

## 1. Introduction

Non-valvular atrial fibrillation (NVAF) is a growing health issue in the aging society [1,2]. Elderly individuals and those with comorbidities have an increased risk of ischemic stroke and vascular events. Although new-generation nonvitamin K antagonists (NOACs) have demonstrated notable convenience and safety in the prevention of cardioembolic strokes [3], clinicians still struggle to achieve intensive medical control in NVAF patients with stroke risk [4,5,6,7,8]. Dabigatran etexilate, a direct thrombin inhibitor, is one NOAC that showed superior efficacy in vascular prevention compared to the traditional vitamin K antagonist, warfarin, when patients take high-dose dabigatran (150 mg twice daily) based on the results of the Randomized Evaluation of Long-Term Anticoagulant Therapy (RE-LY) trial [9]. Another approved low dabigatran dose (110 mg twice daily), which was non-inferior to warfarin regarding efficacy, had safety benefits, with lower rates of major bleeding. Patients with specific physical conditions and comorbidities have different risks of vascular events and bleeding [10,11]. Achieving optimal personalized medical prevention is desirable to balance the benefits and side effects of anticoagulant therapy based on patients’ features. According to a post-hoc analysis of the RE-LY trial data, European label-simulated dabigatran treatment, which recommends adjusting dabigatran dose according to patient’s age, risk of bleeding, or when patients are on verapamil, can achieve superior efficacy and safety compared to warfarin [12]. Researchers validated a prediction model for shared decision-making before starting dabigatran treatment [13,14].

Traditional evaluation tools such as the CHA_2_DS_2_-VASc and HAS-BLED scores have provided concepts of the risk of ischemic stroke and bleeding in patients with NVAF. In recent years, machine learning (ML)-based tools for analyzing data have shown efficacy in identifying the influence and interaction of important factors in the medical context [15,16]. Unlike the popular logistic regression (LGR) method, which shows the binary results for the target variable using a regression model, ML models have the advantage of being able to explore complex hidden rules in massive, disorganized data between predictors and the target variable presenting with diverse phenotype [17,18]. ML methods have shown good performance for prediction and feature selection in cardiovascular, chronic respiratory diseases, myocardial infarction, schizophrenia, tongue cancer, and colorectal cancer [19,20,21,22,23,24]. Under the concept of collective intelligence, an integrated ML feature selection and prediction scheme achieved advanced efficacy in selecting important variables of vascular events and bleeding according to the RE-LY trial database [25].

However, the interpretability of ML is also a growing concern because many models work in “black boxes” [26]. In clinical practice, every patient has a combination of diverse characteristics and underlying diseases that require careful consideration before selecting the appropriate therapy [27]. A transparent and explainable rule is essential to solve these kinds of problems. Patients may have several risk factors for vascular events and bleeding. More comprehensive information about the vascular event and bleeding risk may establish suggestive guidelines to optimize the use of dabigatran.

A ML decision tree algorithm is a tree-based classifier composed of nodes representing selected variables; the division of the tree into prognostic outcomes shows rules regarding the combinations of these variables [28,29]. Decision tree models provide a good structure for presenting medical rules that are similar to the clinician’s decision-making process [30,31]. This study aimed to construct a multi-step ML decision tree scheme that first identified important variables based on ML algorithms and then generated a tree-shaped rule based on the selected important variables according to dataset of the RE-LY trial. The classification and regression tree (CART) has been used in many medical conditions, such as dementia, stroke, influenza infection, and malignancy, because it has good visualizable and explainable information [32,33,34,35] for guiding clinical decision-making. Our study proposes an integrated ML decision-tree scheme to achieve precision medicine in deciding the appropriate dose selection for patients with NVAF.

## 2. Materials and Methods

### 2.1. Study Population

Our research adopted an ML method for secondary analysis based on the dataset of the RE-LY trial. The Fu Jen Catholic University Hospital’s Research Ethics Review Committee examined and approved this study (IRB No. FJUH111180). Since the data solely contained de-identified content, informed permission was waived.

Dabigatran 110 or 150 mg twice daily or an adjusted dose of warfarin were the three treatment options in the RE-LY trial. There were 18113 patients with newly diagnosed arrhythmia and evidence suggesting secondary prevention randomly assigned to receive one kind of anticoagulant with a median follow-up time of approximately two years. A history of severe valvular heart disease, recent stroke, and renal failure was listed as exclusion criteria. A stroke or systemic embolism was the primary outcome, as major bleeding was the primary safety outcome [9]. The RE-LY trial was a multi-center, randomized trial with 99.9% of participants completing follow-up. Data from patients who received dabigatran and finished the RE-LY trial’s follow-up were gathered for this study. Two study subgroups of patients receiving dabigatran 150 mg and 110 mg twice daily were designed.

### 2.2. Method

**Figure 1:**
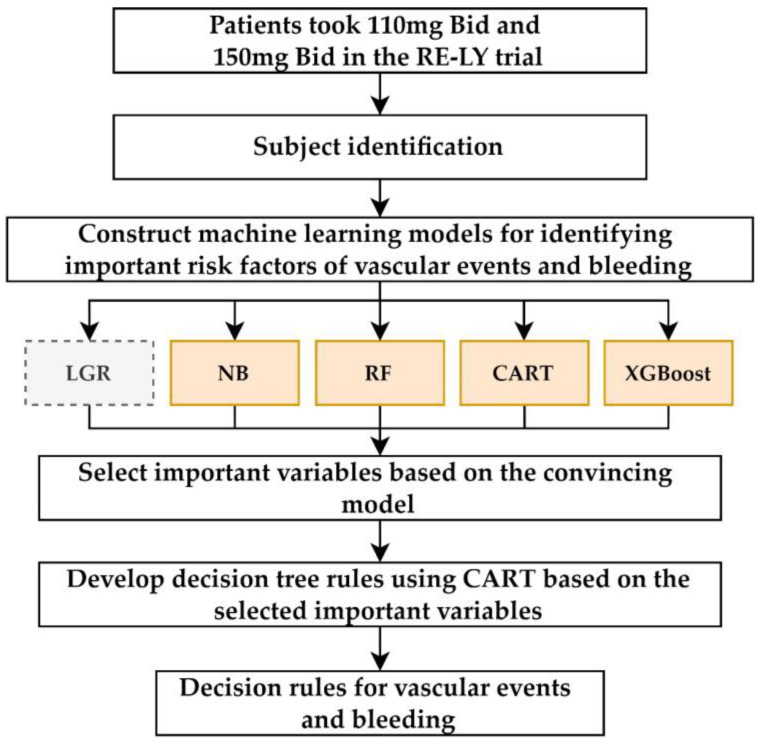
Flowchart of our multi-step machine learning scheme.

We proposed an integrated multi-step ML scheme (Figure 1) to construct a decision-tree model for risk evaluation in patients with NVAF taking different doses of dabigatran. Our protocol applied four ML algorithms: naive Bayes (NB), CART, random forest (RF), and extreme gradient boosting (XGBoost), which have been widely used in various medical informatics applications to select important variables [36,37,38,39,40]. LGR is a classic classification method that uses data fitting to a logistic function to estimate the likelihood of an event occurring [41]. The multivariate LGR model, which uses numerical or categorical predictor variables, was commonly used in medical research and as the benchmark method for comparing the performance of these ML methods. CART was used as the ML decision-tree algorithm to create our rules in the second step.

CART is a binary splitting decision tree method based on the Gini index, which could be a classifier or regression predictive tool with an optimal tree size by applying a cross-validation (CV) procedure [42]. NB is a well-known probabilistic classifier based on Bayes’ theorem with strong independence assumptions between individual features [43]. RF is an ensemble classification method based on a decision tree [44]. Each tree grows to its best set of explanatory variables by the bootstrap resampling technique. The majority vote of these trees decide the model’s final prediction. XGBoost is another tree-based learning algorithm with an interrelated base classifier [45]. Unlike RF, it adjusts the imperfections or inadequacies of the previous model in building new models to accelerate tree construction and prevent overfitting.

First step: Define variables and classify the study subgroups. Our study referred to the recommendations of the American Heart Association and the European Society of Cardiology [1,2]. The personal features, medical history and biochemical data of the patients were defined as predictor variables. The target variable in the 110 mg group was vascular events (stroke, systemic embolism, and vascular death) and the target variable in the 150 mg group was bleeding. The description of our predictive variables (X1–X17) and two target variables (P1, P2) are shown in Supplementary Table S1.

Second step: Construct an ML model to identify important variables of vascular events and bleeding in the two study subgroups. In our study, the dataset was randomly divided into an 80% training dataset for constructing the acceptable model with the best hyperparameter set and a 20% testing dataset for model testing. Our protocol applied a 10-fold CV method to enhance stability for tuning the hyperparameters for the best performance of NB, RF, CART, and XGBoost models based on the area under the receiver operating characteristic (ROC) curve (AUC) values. Tenfold CV involves the modifying the validation dataset ten times to generate the adequate validation performance [46]. The performance of the model was compared according to the AUC value superior to the result of LGR. We built each method with R software (http://www.R-project.org; accessed on 27 October 2022; https://www.rstudio.com/products/rstudio/; accessed on 27 October 2022) with the required installed packages. NB, RF, CART, and XGBoost were implemented, respectively. The “caret” R package version 6.0–90 was used for each method to generate the importance values of individual variables. Averaging the importance values of predictive variables in the selected models, we chose the ten most important variables for vascular events and bleeding in the 110 mg and 150 mg groups, respectively, as nodes in the decision tree model.

Third step: Develop decision rules using CART based on the selected important variables. Using the same model construction process in the previous step, the 10-fold CV was used to train the CART model. The CART decision tree model with the best performance was used to establish tree-shaped rules composed of the 10 selected variables for predicting vascular events and bleeding in the 110 mg and 150 mg groups, respectively.

In the last step, the rules derived from the important variables in our scheme were used to clarify what clinicians should avoid when selecting the dabigatran dose for NVAF patients. The discussion of these rules may provide us with graphical information on the precise dose of dabigatran.

## 3. Results

Our study recruited 5869 patients who took 110 mg dabigatran twice daily and 5933 patients who took 150 mg dabigatran twice. Table 1 shows the demographic data of the 110 mg and 150 mg groups. Vascular events and bleeding occurred in 185 (3.15%) and 1189 (20.04%) patients within the first year of follow-up in the 110 mg and 150 mg groups, respectively.

**Figure 2:**
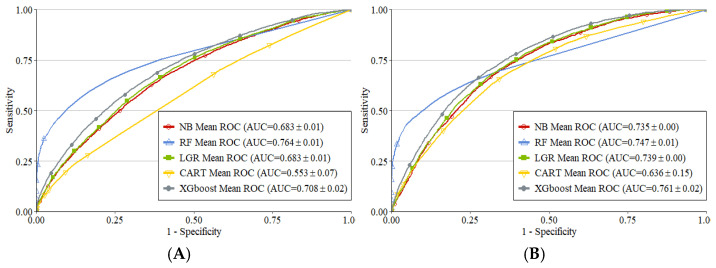
ROC curves of the five methods for predicting vascular events and bleeding. (**A**) Vascular events in the 110 mg group; (**B**) Bleeding in the 150 mg group.

**Figure 3:**
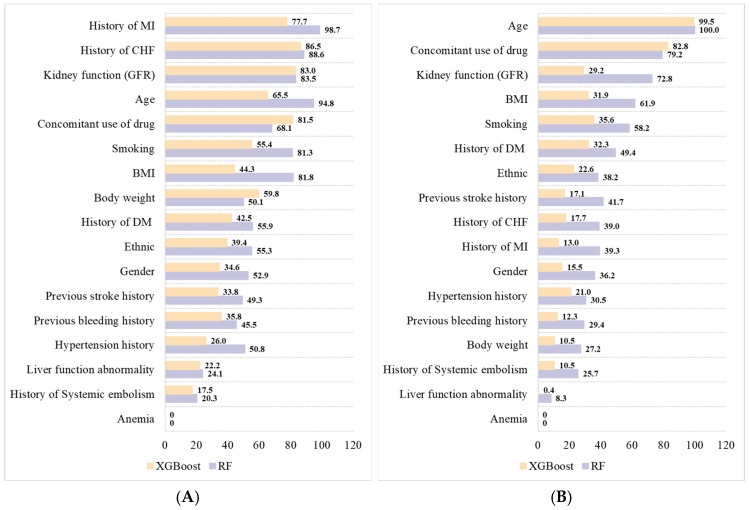
Average importance values of variables in the RF and XGBoost models based on 10-fold cross-validation. (**A**) Variables predicting vascular events in the 110 mg group; (**B**) Variables predicting bleeding in the 150 mg group.

Table 2 shows the performances of LGR, NB, RF, CART, and XGBoost methods in predicting vascular events and bleeding in each group with their best hyperparameters. From the table, RF and XGBoost presented advanced predictive performance for vascular events in the 110 mg group and bleeding in the 150 mg group according to their AUC values. The ROC curves of the five methods in predicting vascular events and bleeding are shown in Figure 2. RF and XGBoost were selected as the modes because NB and CART showed inferior performance to LGR. Figure 3 shows the average importance values of variables in the RF and XGBoost models derived from the 10-fold CV. The top ten important variables in the two groups based on the average values in RF and XGBoost models are presented in Table 3.

**Figure 4:**
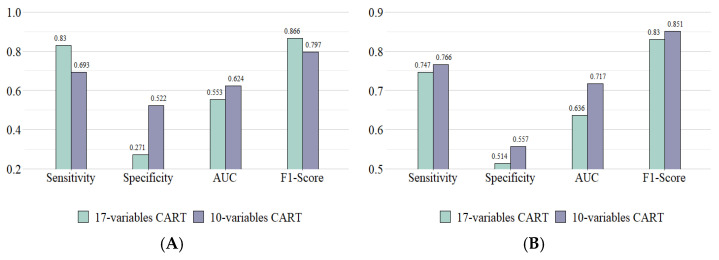
The performance of the CART model after adjusting variables according to our scheme. (**A**) CART predicting vascular events in the 110 mg group; (**B**) CART predicting bleeding in the 150 mg group.

The top ten important variables were used as inputs for rebuilding the CART decision-tree models. Figure 4 shows the performance of the CART model after adjusting for variables. The reconstructed CART model with 10 important variables generated better AUC values predicting vascular events (0.624) and bleeding (0.717) than that of the CART model with all 17 variables. After removing some variables, we obtained a more balanced performance and better AUC values in the CART models after adjusting for variables according to our feature selection scheme.

**Figure 5:**
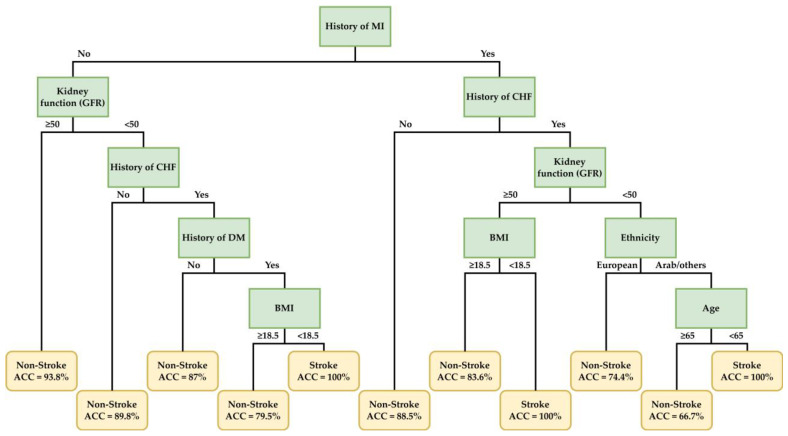
Decision rules for predicting vascular events in patients taking dabigatran 110 mg twice daily based on important clinical factors of the best CART model.

**Figure 6:**
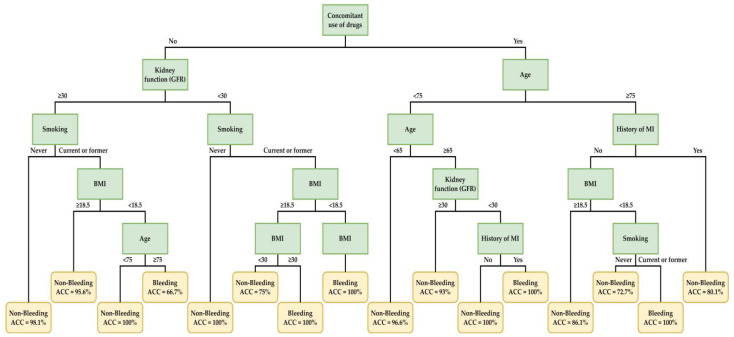
Decision rules for predicting bleeding in patients taking dabigatran 150 mg twice daily based on important clinical factors of the best CART model.

Interpreting the CART-generated rules in the 110 mg group, patients with a combination of a history of congestive heart failure (CHF), MI, diabetes mellitus (DM), kidney function abnormality, ethnicity, body mass index (BMI), and age feature had a tendency towards vascular events (Figure 5). Patients with a combination of the concomitant use of drugs, kidney function abnormality, smoking habit, history of MI, BMI, and age features had a high risk of bleeding in the 150 mg group (Figure 6). Details regarding the decision rules predicting vascular events in the dabigatran 110 mg group and bleeding in the dabigatran 150 mg group are provided in Table 4 and Table 5, respectively. The accuracy (ACC) demonstrated in these tables and figures is defining as below.

$$Accuracy=\frac{True\;positive+True\;negative}{True\;positive+True\;negative+False\;positive+False\;negative}$$

## 4. Discussion

To the best of our knowledge, our study is the first to apply an ML-based decision-tree model to NVAF patients taking dabigatran. Clinicians need to determine the balance point between preventing vascular events and avoiding bleeding. Although NOACs possess prior safety than warfarin, approximately 9–39% of patients had off-label low-dose NOACs in real-world research for concern about risk of bleeding or other side effects [27,47,48]. Many studies have identified risk factors and evaluation systems according to the LGR results [1,2]. An integrated ML scheme helped to identify important risk factors for vascular events and bleeding by analyzing the RE-LY trial data [25]. This kind ML scheme had more promising performance when we targeted vascular events in the dabigatran 110 mg group and bleeding in the dabigatran 150 mg group. RF and XGBoost were the best ML models in our analysis (Table 3).

Among ML techniques, decision tree models are more able to demonstrate clear rules. Like in most clinical settings, this method recursively partitions the data into subsets based on the values of the input features. The final work generates a tree-like model that can be used to make predictions by traversing the tree from the root to a leaf node, and the decision rules are the conditions at each node that determine which branch to follow. This allows the establishment of personalized suggestions and protocols to achieve precision medicine. CART is a popular tool for creating tree-shaped medical rules with realistic visualization and interpretability. It is a classifier and decision tree algorithm, but it showed inferior prediction performance in the second step of our study. We improved its AUC value by selecting fewer important variables with our integrated ML feature selecting scheme. Thus, we obtained explainable tree rules with acceptable predictive values. Figure 5 shows the decision rules for predicting vascular events, with five layers of nodes and 11 leaves. Figure 6 shows the decision rules for predicting bleeding, and it had five layers of nodes and 16 leaves. According to these suggestive medical rules based on important variables, clinicians may have a more detailed view and assistance in making decisions.

In the tree-shaped rule, the most important variables may be used to classify patients into high-risk and low-risk groups. We may notice that it is challenging to predict patients who may be free from vascular events in the next year when taking dabigatran 110 mg. Most rules indicating freedom from events demonstrated accuracy of 60–90%. However, we may surmise that 110 mg dabigatran was a relatively acceptable dose in patients without a history of MI, CHF, and kidney function abnormality. The risk of stroke and vascular events in patients with NVAF is determined by coexisting systemic diseases and age. The annual stroke risk was approximately 2–3% in low-risk patients based on the CHA_2_DS_2_-VASc score. In many national cohort studies, most clinicians prescribed off-label reduced doses of NOACs in consideration of age, body weight, kidney function abnormality, and medical history. Our research offers another viewpoint that may help in the prescription of reduced-dose NOACs to patients with a low risk of vascular events. However, more critically, the decision tree demonstrated that patients with certain variables had an extremely high risk of vascular events while taking 110 mg dabigatran twice daily.

MI and CHF are important comorbidities and risk factors in NVAF patients, which greatly influence stroke and vascular death. This might be the result of different vascular risk factors, such as hypertension, DM, smoking, and dyslipidemia. In our tree model, NVAF patients with a history of MI and CHF had a higher risk of vascular events when taking dabigatran 110 mg twice daily. Furthermore, they had a greater risk of vascular events when they had low BMI < 18.5 kg/m^2^ or kidney function abnormality (EGRF < 50 mL/min/1.73 m^2^). For those without a history of MI, a combination of CHF, history of DM, and kidney function abnormality was associated with a high risk of vascular events when on dabigatran 110 mg, especially in underweight persons (BMI < 18.5 kg/m^2^). Thus, applying intensive medical control and a standard dose of dabigatran 150 mg, if tolerable, is a more reasonable choice for clinicians.

When we consider safety with dabigatran 150 mg, Figure 6 shows the rules related to the high risk of bleeding. The concomitant use of certain drugs and older age (age ≥75 years) were the top factors that increased the risk of bleeding, especially when patients were underweight and smoker. For patients aged 65–74 years, dabigatran 150 mg might be risky if there is the concomitant use of certain drugs and kidney function abnormality (EGRF < 30 mL/min/1.73 m^2^). The combination of kidney function abnormality (EGRF < 30 mL/min/1.73 m^2^) and smoking habits also increased bleeding risk, regardless of BMI. Otherwise, the rule also indicated that elderly patients (age ≥75 years) with smoking habits and underweight stature tended to bleed when taking 150 mg dabigatran. Adjusting the dose of dabigatran may be considered in these patients.

The primary prevention of stroke in NVAF patients has been a prevalent issue in recent years due to the progression of NOACs. Intensive risk-factor control and the early application of anticoagulants in patients prone to vascular events are emphasized. This study provides an integrated multi-step ML scheme to assist in selecting the appropriate dose of dabigatran. In general, it is crucial to first determine if the patients have a history of MI, CHF, or kidney function abnormality with regard to the risk of vascular events, and if there is the contaminant use of drugs, kidney function abnormality, older age, and smoking habit with regard to the risk of bleeding. To confirm the most appropriate dose of dabigatran for the patient, clinicians may check the detailed classification information in our tree model.

Our model might be extended to more medical issues with its good prediction ability and convenience. However, dose adjustments and patient selection guidelines may take time before confirmation according to cohort studies and expert opinion. Our integrated ML scheme with adequate detailed data should provide clinicians with personalized decision support and recommendations. Moreover, ML techniques have the advantage of remodeling and may improve their prediction efficacy with new dataset. In this way, experts may reinspect and utilize our model to construct medical guidelines.

## 5. Limitation

There are some limitations to this study. First, our study was a post-hoc analysis of the RE-LY trial and excluded patients with recent ischemic stroke, and the participants’ CHA_2_DS_2_-VASc scores were 2.1 ± 1.1, which might be different from the real-world situation. Our results might be inappropriate for patients in the acute stage of stroke or other extremely high-risk conditions. Second, we conducted a subgroup analysis to evaluate bleeding and vascular event risk. This procedure decreased the dataset size, and we did not perform CV to confirm the influence of different choices. However, the prediction performance value remained consistent in the two subgroups, and we performed 10-fold nested CV to enhance stability and avoid overfitting. The application of anticoagulant agents in NVAF patients with risk of vascular events and the choice of NOACs as first-line drugs have been the consensus management modality. We only focused on the dose selection for dabigatran but did not compare the doses with warfarin. However, the limited dataset and lack of external validation with real-world information restrict the consistency of our decision tree rules. Further remodeling our model with broad information is indicated before the utilization.

## 6. Conclusions

This study used an integrated ML-based decision tree scheme to predict vascular events and bleeding when NVAF patients took different doses of dabigatran. According to the integrated ML feature-selection tool, we utilized the performance of the CART method, which should provide clinicians with detailed and interpretable information. Our tree-shaped rules should demonstrate the interaction between factors and the most risk-prone combinations of variables. Appropriate dose selection of NOACs for balancing benefits and side effects is an art in medicine. Our results may help clinicians achieve the optimal treatment for NVAF with different characteristics.

## Figures and Tables

**Table 1:** Clinical and demographic characteristics of the 110 mg and 150 mg groups.

Variables	110 mg		150 mg	
	Category	N (%)	Category	N (%)
Gender	Male	3770 (64.24%)	Male	3748 (63.17%)
	Female	2099 (35.76%)	Female	2185 (36.83%)
Age	<65	977 (16.65%)	<65	1005 (16.94%)
	≥65 and <75	2606 (44.40%)	≥65 and <75	2517 (42.42%)
	≥75	2286 (38.95%)	≥75	2411 (40.64%)
BMI	<18.5	61 (1.04%)	<18.5	62 (1.05%)
	≥18.5 and <30	3764 (64.13%)	≥18.5 and <30	3824 (64.45%)
	≥30	2044 (34.83%)	≥30	2047 (34.50%)
Body weight	<60	554 (9.44%)	<60	544 (9.17%)
	≥60	5315 (90.56%)	≥60	5389 (90.83%)
Ethnicity	Arab/others	1798 (30.64%)	Arab/others	1796 (30.27%)
	European	4071 (69.36%)	European	4137 (69.73%)
Hypertension history	Yes	1248 (21.26%)	Yes	1254 (21.14%)
	No	4621 (78.74%)	No	4679 (78.86%)
Kidney function (GFR)	<30	13 (0.22%)	<30	32 (0.54%)
	≥30 and <50	1113 (18.96%)	≥30 and <50	1132 (19.08%)
	≥50	4743 (80.81%)	≥50	4769 (80.38%)
Previous stroke history	Yes	1166 (19.87%)	Yes	1199 (20.21%)
	No	4703 (80.13%)	No	4734 (79.79%)
Previous bleeding history	Yes	386 (6.58%)	Yes	388 (6.54%)
	No	5483 (93.42%)	No	5545 (93.46%)
Concomitant use of drug	Yes	1429 (24.35%)	Yes	1415 (23.85%)
	No	4440 (75.65%)	No	4518 (76.15%)
History of MI	Yes	987 (16.82%)	Yes	995 (16.77%)
	No	4882 (83.18%)	No	4938 (83.23%)
History of DM	Yes	1376 (23.45%)	Yes	1362 (22.96%)
	No	4493 (76.55%)	No	4571 (77.04%)
History of CHF	Yes	2069 (35.25%)	Yes	2056 (34.65%)
	No	3800 (64.75%)	No	3877 (65.35%)
Smoking	Never	2866 (48.83%)	Never	2915 (49.13%)
	Current	429 (7.31%)	Current	438 (7.38%)
	Former	2574 (43.86%)	Former	2580 (43.49%)
History of SE	Yes	150 (2.56%)	Yes	156 (2.63%)
	No	5719 (97.44%)	No	5777 (97.37%)
Liver function abnormality	Yes	49 (0.83%)	Yes	35 (0.59%)
	No	5820 (99.17%)	No	5898 (99.41%)
Anemia	Yes	20 (0.34%)	Yes	10 (0.17%)
	No	5849 (99.66%)	No	5923 (99.83%)
Vascular events	Yes	185 (3.15%)	-	-
	No	5684 (96.85%)	-	-
Bleeding	-	-	Yes	1189 (20.04%)
	-	-	No	4744 (79.96%)

Abbr.: BMI—body mass index; GFR—glomerular filtration rate; MI—myocardial infarction; DM—diabetes mellitus; CHF—congestive heart failure; SE—systemic embolism.

**Table 2:** Performance of the LGR, NB, RF, XGBoost models in predicting vascular events in the 110 mg group and bleeding in the 150 mg group.

Methods	Sensitivity Mean (SD)	Specificity Mean (SD)	AUC Mean (SD)	F1-Score Mean (SD)
**(A) Predicting vascular events in dabigatran 110 mg group**				
NB	0.606 (0.05)	0.680 (0.05)	0.683 (0.01)	0.740 (0.04)
**RF**	0.840 (0.03)	0.592 (0.03)	**0.764 (0.01)**	0.895 (0.02)
LGR	0.608 (0.05)	0.674 (0.05)	0.683 (0.01)	0.741 (0.04)
CART	0.830 (0.19)	0.271 (0.29)	0.553 (0.07)	0.866 (0.10)
**XGBoost**	0.665 (0.06)	0.650 (0.05)	**0.708 (0.02)**	0.782 (0.04)
**(B) Predicting bleeding in dabigatran 150 mg group**				
NB	0.661 (0.04)	0.707 (0.04)	0.735 (0.00)	0.785 (0.03)
**RF**	0.860 (0.05)	0.555 (0.05)	**0.747 (0.01)**	0.908 (0.03)
LGR	0.640 (0.04)	0.731 (0.04)	0.739 (0.00)	0.770 (0.03)
CART	0.747 (0.15)	0.514 (0.28)	0.636 (0.15)	0.830 (0.09)
**XGBoost**	0.676 (0.04)	0.724 (0.04)	**0.761 (0.02)**	0.796 (0.03)

Abbr.: SD—standard deviation.

**Table 3:** Top ten important variables for predicting vascular events and bleeding.

Average Ranking of Variables	Variable of Prediction of Vascular Events in 110 mg Group	Average Importance (%)	Variable of Prediction of Bleedings in 150 mg Group	Average Importance (%)
1	History of MI	88.2	Age	99.8
2	History of CHF	87.5	Concomitant use of drug	81.0
3	Kidney function	83.2	Kidney function	51.0
4	Age	80.2	BMI	46.9
5	Concomitant use of drug	74.8	Smoking	46.9
6	Smoking	68.4	History of DM	40.9
7	BMI	63.0	Ethnic	30.4
8	Body weight	54.9	Previous stroke history	29.4
9	History of DM	49.2	History of CHF	28.4
10	Ethnic	47.4	History of MI	26.2

Abbr.: CHF—congestive heart failure, MI—myocardial infarction, BMI—body mass index, DM—diabetes mellitus.

**Table 4:** Summarized decision rules of the combinations of important variables for predicting vascular events.

Rules No.	Combinations of Clinical Factors	Stroke (Yes/No)	Accuracy
1	History of MI (No) + Kidney function (≥50)	No	93.8%
2	History of MI (No) + Kidney function (<50) + History of CHF (No)	No	89.8%
3	History of MI (No) + Kidney function (<50) + History of CHF (Yes) + History of DM (No)	No	87%
4	History of MI (No) + Kidney function (<50) + History of CHF (Yes) + History of DM (Yes) + BMI (≥18.5)	No	79.5%
5	History of MI (No) + Kidney function (<50) + History of CHF (Yes) + History of DM (Yes) + BMI (<18.5)	Yes	100%
6	History of MI (Yes) + History of CHF (No)	No	88.5%
7	History of MI (Yes) + History of CHF (Yes) + Kidney function (≥50) + BMI (≥18.5)	No	83.6%
8	History of MI (Yes) + History of CHF (Yes) + Kidney function (≥50) + BMI (<18.5)	Yes	100%
9	History of MI (Yes) + History of CHF (Yes) + Kidney function (<50) + Ethnicity (European)	No	74.4%
10	History of MI (Yes) + History of CHF (Yes) + Kidney function (<50) + Ethnicity (Arab/others) + Age (≥65)	No	66.7%
11	History of MI (Yes) + History of CHF (Yes) + Kidney function (<50) + Ethnicity (Arab/others) + Age (<65)	Yes	100%

**Table 5:** Summarized decision rules of combinations of important variables for predicting bleeding.

Rules No.	Combinations of Clinical Factors	Bleeding (Yes/No)	Accuracy
1	Concomitant use of drugs (No) + Kidney function (≥30) + Smoking (Never)	No	98.1%
2	Concomitant use of drugs (No) + Kidney function (≥30) + Smoking (Current or former) + BMI (≥18.5)	No	95.6%
3	Concomitant use of drugs (No) + Kidney function (≥30) + Smoking (Current or former) + BMI (<18.5) + Age (<75)	No	100%
4	Concomitant use of drugs (No) + Kidney function (≥30) + Smoking (Current or former) + BMI (<18.5) + Age (≥75)	Yes	66.7%
5	Concomitant use of drugs (No) + Kidney function (<30) + Smoking (Never)	No	100%
6	Concomitant use of drugs (No) + Kidney function (<30) + Smoking (Current or former) + BMI (18.5–29.9)	No	75%
7	Concomitant use of drugs (No) + Kidney function (<30) + Smoking (Current or former) + BMI (≥30)	Yes	100%
8	Concomitant use of drugs (No) + Kidney function (<30) + Smoking (Current or former) + BMI (<18.5)	Yes	100%
9	Concomitant use of drugs (Yes) + Age (<65)	No	96.6%
10	Concomitant use of drugs (Yes) + Age (65–74) + Kidney function (≥30)	No	93%
11	Concomitant use of drugs (Yes) + Age (65–74) + Kidney function (<30) + History of MI (No)	No	100%
12	Concomitant use of drugs (Yes) + Age (65–74) + Kidney function (<30) + History of MI (Yes)	Yes	100%
13	Concomitant use of drugs (Yes) + Age (≥75) + History of MI (No) + BMI (≥18.5)	No	86.1%
14	Concomitant use of drugs (Yes) + Age (≥75) + History of MI (No) + BMI (<18.5) + Smoking (Never)	No	72.7%
15	Concomitant use of drugs (Yes) + Age (≥75) + History of MI (No) + BMI (<18.5) + Smoking (Current or former)	Yes	100%
16	Concomitant use of drugs (Yes) + Age (≥75) + History of MI (Yes)	No	80.1%

## Data Availability

The data are available through application to Boehringer-Ingelheim on the research data sharing platform (https://vivli.org/; accessed on 28 July 2021). Restrictions apply to the availability of these data, which were used under license for this study. Analysis codes are available on request to the corresponding author due to privacy/ethical restrictions.

## Supplementary Materials

The following supporting information can be downloaded at: https://www.mdpi.com/article/10.3390/ijerph20032359/s1, Table S1: Predictive variables (X1–X17), two target variables (P1, P2), and their descriptions in this study.

## References

[1] January C.T., Wann L.S., Calkins H., Chen L.Y., Cigarroa J.E., Cleveland J.C., Ellinor P.T., Ezekowitz M.D., Field M.E., Furie K.L. (2019). 2019 AHA/ACC/HRS Focused Update of the 2014 AHA/ACC/HRS Guideline for the Management of Patients With Atrial Fibrillation: A Report of the American College of Cardiology/American Heart Association Task Force on Clinical Practice Guidelines and the Heart Rhythm Society. J. Am. Coll. Cardiol..

[2] Hindricks G., Potpara T., Dagres N., Arbelo E., Bax J.J., Blomström-Lundqvist C., Boriani G., Dan G.A., Dilaveris P.E., Fauchier L. (2021). 2020 ESC Guidelines for the Diagnosis and Management of Atrial Fibrillation Developed in Collaboration with the European Association for Cardio-Thoracic Surgery (EACTS). Eur. Heart J..

[3] Ntaios G., Papavasileiou V., Diener H.C., Makaritsis K., Michel P. (2012). Nonvitamin-K-antagonist oral anticoagulants in patients with atrial fibrillation and previous stroke or transient ischemic attack: A systematic review and meta-analysis of randomized controlled trials. Stroke.

[4] Ntaios G., Papavasileiou V., Makaritsis K., Vemmos K., Michel P., Lip G.Y.H. (2017). Real-World Setting Comparison of Nonvitamin-K Antagonist Oral Anticoagulants Versus Vitamin-K Antagonists for Stroke Prevention in Atrial Fibrillation: A Systematic Review and Meta-Analysis. Stroke.

[5] Chan Y.-H., See L.-C., Tu H.-T., Yeh Y.-H., Chang S.-H., Wu L.-S., Lee H.-F., Wang C.-L., Kuo C.-F., Kuo C.-T. (2018). Efficacy and Safety of Apixaban, Dabigatran, Rivaroxaban, and Warfarin in Asians With Nonvalvular Atrial Fibrillation. J. Am. Heart Assoc..

[6] Lip G.Y.H., Keshishian A., Li X., Hamilton M., Masseria C., Gupta K., Luo X., Mardekian J., Friend K., Nadkarni A. (2018). Effectiveness and safety of oral anticoagulants among nonvalvular atrial fibrillation patients: The ARISTOPHANES study. Stroke.

[7] Chan Y.H., Lee H.F., See L.C., Tu H.T., Chao T.F., Yeh Y.H., Wu L.S., Kuo C.T., Chang S.H., Lip G.Y.H. (2019). Effectiveness and safety of four direct oral anticoagulants in Asian patients with nonvalvular atrial fibrillation. Chest.

[8] Ajabnoor A.M., Zghebi S.S., Parisi R., Ashcroft D.M., Rutter M.K., Doran T., Carr M.J., Mamas M.A., Kontopantelis E. (2022). Incidence of nonvalvular atrial fibrillation and oral anticoagulant prescribing in England, 2009 to 2019: A cohort study. PLoS Med..

[9] Connolly S.J., Ezekowitz M.D., Yusuf S., Eikelboom J., Oldgren J., Parekh A., Wang S. (2009). Dabigatran versus warfarin in patients with atrial fibrillation. N. Engl. J. Med..

[10] Chen L.Y., Norby F.L., Chamberlain A.M., MacLehose R.F., Bengtson L.G.S., Lutsey P.L., Alonso A. (2019). CHA_2_DS_2_-VASc Score and Stroke Prediction in Atrial Fibrillation in Whites, Blacks, and Hispanics. Stroke.

[11] Chang G., Xie Q., Ma L., Hu K., Zhang Z., Mu G., Cui Y. (2020). Accuracy of HAS-BLED and other bleeding risk assessment tools in predicting major bleeding events in atrial fibrillation: A network meta-analysis. J. Thromb. Haemost..

[12] Lip G.Y., Clemens A., Noack H., Ferreira J., Connolly S.J., Yusuf S. (2014). Patient outcomes using the European label for dabigatran. A post-hoc analysis from the RE-LY database. Thromb. Haemost..

[13] Stam-Slob M.C., Connolly S.J., van der Graaf Y., van der Leeuw J., Dorresteijn J.A., Eikelboom J.W., Peters R.J., Alings M., Visseren F.L. (2019). Individual treatment effect estimation of 2 doses of Dabigatran on stroke and major bleeding in atrial fibrillation: Results from the RE-LY trial. Circulation.

[14] Reinhardt S.W., Desai N.R., Tang Y., Jones P.G., Ader J., Spertus J.A. (2021). Personalizing the decision of dabigatran versus warfarin in atrial fibrillation: A secondary analysis of the Randomized Evaluation of Long-term anticoagulation therapY (RE-LY) trial. PLoS ONE.

[15] Liu Y., Chen P.-H., Krause J., Peng L. (2019). How to Read Articles That Use Machine Learning: Users’ Guides to the Medical Literature. JAMA.

[16] Kamel H., Navi B.B., Parikh N.S., Merkler A.E., Okin P.M., Devereux R.B., Weinsaft J.W., Kim J., Cheung J.W., Kim L.K. (2020). Machine Learning Prediction of Stroke Mechanism in Embolic Strokes of Undetermined Source. Stroke.

[17] Ting W.-C., Chang H.-R., Chang C.-C., Lu C.-J. (2020). Developing a Novel Machine Learning-Based Classification Scheme for Predicting SPCs in Colorectal Cancer Survivors. Appl. Sci..

[18] Wu T.-E., Chen H.-A., Jhou M.-J., Chen Y.-N., Chang T.-J., Lu C.-J. (2021). Evaluating the Effect of Topical Atropine Use for Myopia Control on Intraocular Pressure by Using Machine Learning. J. Clin. Med..

[19] Al’Aref S.J., Anchouche K., Singh G., Slomka P.J., Kolli K.K., Kumar A., Pandey M., Maliakal G., van Rosendael A.R., Beecy A.N. (2019). Clinical applications of machine learning in cardiovascular disease and its relevance to cardiac imaging. Eur. Heart. J..

[20] Shah W., Aleem M., Iqbal M.A., Islam M.A., Ahmed U., Srivastava G., Lin J.C. (2021). A Machine-Learning-Based System for Prediction of Cardiovascular and Chronic Respiratory Diseases. J. Healthc. Eng..

[21] Than M.P., Pickering J.W., Sandoval Y., Shah A.S.V., Tsanas A., Apple F.S., Blankenberg S., Cullen L., Mueller C., Neumann J.T. (2019). Machine learning to predict the likelihood of acute myocardial infarction. Circulation.

[22] Ke P.F., Xiong D.S., Li J.H., Pan Z.L., Zhou J., Li S.J., Song J., Chen X.Y., Li G.X., Chen J. (2021). An integrated machine learning framework for a discriminative analysis of schizophrenia using multi-biological data. Sci. Rep..

[23] Alabi R.O., Elmusrati M., Sawazaki-Calone I., Kowalski L.P., Haglund C., Coletta R.D., Mäkitie A.A., Salo T., Leivo I., Almangush A. (2019). Machine learning application for prediction of locoregional recurrences in early oral tongue cancer: A Web-based prognostic tool. Virchows Arch..

[24] Yu H., Huang T., Feng B., Lyu J. (2022). Deep-learning model for predicting the survival of rectal adenocarcinoma patients based on a surveillance, epidemiology, and end results analysis. BMC Cancer.

[25] Huang Y.-C., Cheng Y.-C., Jhou M.-J., Chen M., Lu C.-J. (2022). Important Risk Factors in Patients with Nonvalvular Atrial Fibrillation Taking Dabigatran Using Integrated Machine Learning Scheme—A Post Hoc Analysis. J. Pers. Med..

[26] Rudin S. (2019). Stop Explaining Black Box Machine Learning Models for High Stakes Decisions and Use Interpretable Models Instead. Nat. Mach. Intell..

[27] Steinberg B.A., Shrader P., Thomas L., Ansell J., Fonarow G.C., Gersh B.J., Kowey P.R., Mahaffey K.W., Naccarelli G., Reiffel J. (2016). Off-Label Dosing of Non-Vitamin K Antagonist Oral Anticoagulants and Adverse Outcomes: The ORBIT-AF II Registry. J. Am. Coll. Cardiol..

[28] Anooj P.K.N. (2011). Clinical decision support system: Risk level prediction of heart disease using weighted fuzzy rules and decision tree rules. Cent. Eur. J. Comput. Sci..

[29] Persi Pamela I., Gayathri P., Jaisankar N. (2013). A fuzzy optimization technique for the prediction of coronary heart disease using decision tree. Int. J. Eng. Technol..

[30] Shih C.-C., Lu C.-J., Chen G.-D., Chang C.-C. (2020). Risk Prediction for Early Chronic Kidney Disease: Results from an Adult Health Examination Program of 19,270 Individuals. Int. J. Environ. Res. Public Health.

[31] Chang C.-C., Yeh J.-H., Chiu H.-C., Chen Y.-M., Jhou M.-J., Liu T.-C., Lu C.-J. (2022). Utilization of Decision Tree Algorithms for Supporting the Prediction of Intensive Care Unit Admission of Myasthenia Gravis: A Machine Learning-Based Approach. J. Pers. Med..

[32] Zimmerman R.K., Balasubramani G.K., Nowalk M.P., Eng H., Urbanski L., Jackson M.L., Jackson L.A., McLean H.Q., Belongia E.A., Monto A.S. (2016). Classification and Regression Tree (CART) analysis to predict influenza in primary care patients. BMC Infect. Dis..

[33] Bivard A., Levi C., Lin L., Cheng X., Aviv R., Spratt N.J., Lou M., Kleinig T., O’Brien B., Butcher K. (2017). Validating a Predictive Model of Acute Advanced Imaging Biomarkers in Ischemic Stroke. Stroke.

[34] Cui X., Heuvelmans M.A., Sidorenkov G., Zhao Y., Fan S., Groen H.J.M., Dorrius M.D., Oudkerk M., de Bock G.H., Vliegenthart R. (2021). A contrast-enhanced-CT-based classification tree model for classifying malignancy of solid lung tumors in a Chinese clinical population. J. Thorac. Dis..

[35] Makino K., Lee S., Bae S., Chiba I., Harada K., Katayama O., Shinkai Y., Shimada H. (2021). Development and validation of new screening tool for predicting dementia risk in community-dwelling older Japanese adults. J. Transl. Med..

[36] Chang C.-C., Yeh J.-H., Chen Y.-M., Jhou M.-J., Lu C.-J. (2021). Clinical Predictors of Prolonged Hospital Stay in Patients with Myasthenia Gravis: A Study Using Machine Learning Algorithms. J. Clin. Med..

[37] Chiu Y.-L., Jhou M.-J., Lee T.-S., Lu C.-J., Chen M.-S. (2021). Health Data-Driven Machine Learning Algorithms Applied to Risk Indicators Assessment for Chronic Kidney Disease. Risk Manag. Healthc. Policy.

[38] Wu C.-W., Shen H.-L., Lu C.-J., Chen S.-H., Chen H.-Y. (2021). Comparison of Different Machine Learning Classifiers for Glaucoma Diagnosis Based on Spectralis OCT. Diagnostics.

[39] Liao P.-C., Chen M.-S., Jhou M.-J., Chen T.-C., Yang C.-T., Lu C.-J. (2022). Integrating Health Data-Driven Machine Learning Algorithms to Evaluate Risk Factors of Early Stage Hypertension at Different Levels of HDL and LDL Cholesterol. Diagnostics.

[40] Sun C.-K., Tang Y.-X., Liu T.-C., Lu C.-J. (2022). An Integrated Machine Learning Scheme for Predicting Mammographic Anomalies in High-Risk Individuals Using Questionnaire-Based Predictors. Int. J. Environ. Res. Public Health.

[41] Peng C.-Y.J., Lee K.L., Ingersoll G.M. (2002). An introduction to logistic regression analysis and reporting. J. Educ. Res..

[42] Breiman L., Friedman J.H., Olshen R.A., Stone C.J. (1984). Classification and Regression Trees. Biometrics.

[43] Lewis D.D. (1998). Naive (Bayes) at forty: The independence assumption in information retrieval. Machine Learning: ECML-98.

[44] Breiman L. (2001). Random Forests. Mach. Learn..

[45] Chen T., Guestrin C. XGBoost: A Scalable Tree Boosting System. Proceedings of the 22nd ACM SIGKDD International Conference on Knowledge Discovery and Data Mining.

[46] Ronchetti E., Field C., Blanchard W. (1997). Robust linear model selection by cross-validation. J. Am. Stat. Assoc..

[47] Arbel R., Sergienko R., Hammerman A., Greenberg-Dotan S., Batat E., Avnery O., Ellis M.H. (2019). Effectiveness and safety of off-label dose-reduced direct oral anticoagulants in atrial fibrillation. Am. J. Med..

[48] Chan Y.-H., Chao T.-F., Chen S.-W., Lee H.-F., Yeh Y.-H., Huang Y.-C., Chang S.-H., Kuo C.-T., Lip G.Y.H., Chen S.-A. (2020). Off-label dosing of non-vitamin K antagonist oral anticoagulants and clinical outcomes in Asian patients with atrial fibrillation. Heart Rhythm..

