# Supplementary material for: Integrated Machine Learning Decision Tree Model for Risk Evaluation in Patients with Non-Valvular Atrial Fibrillation When Taking Different Doses of Dabigatran
Source: Int J Environ Res Public Health. 2023 Jan 29;20(3):2359. doi: 10.3390/ijerph20032359 (PMC9915180; doi:10.3390/ijerph20032359)
Supplement: Supplementary file 1 [file ijerph-20-02359-s001.zip › ijerph-2156031-supplementary.pdf]

Supplementary Table S1. Predictive variables (X1-X17), two target variables (P1, P2), and their descriptions in this study.

	Variables	Description	Units
X1	Gender	**1**: Male; **2**: Female	-
X2	Age	**1**: <65; **2**: ≥65 & <75; **3**: ≥75	year-old
X3	BMI	**1**: <18.5; **2**: ≥18.5 & <30; **3**: ≥30	kg/m^2
X4	Body weight	**1**: <60; **2**: ≥60	kg
X5	Ethnicity	**1**: Arab/others; **2**: European	-
X6	Hypertension history	**1**: Record of hypertension required medical treatment; **2**: No	-
X7	Kidney function (GFR)	**1**: <30; **2**: ≥30 & <50; **3**: ≥50	$ml/min/1.73m^2$
X8	Previous stroke history	**1**: History of stroke or TIA; **2**: No	-
X9	Previous bleeding history	**1**: History of bleeding; **2**: No	-
X10	Concomitant use of drug	**1**: Concomitant use of verapamil, dilteazam, anti-thrombotic agent, NSAID, or COX inhibitor; **2**: No	-
X11	History of MI	**1**: History of MI; **2**: No	-
X12	History of DM	**1**: History of DM; **2**: No	-
X13	History of CHF	**1**: Medical history of CHF or heart echo revealed ejection fraction <40%; **2**: No	-
X14	Smoking	**1**: Never; **2**: Current smoker; **3**: Former history	-
X15	History of systemic embolism*	**1**: History of systemic embolism; **2**: no	-
X16	Liver function abnormality*	**1**: Present of liver function abnormality; **2**: No	-
X17	Anemia	**1**: Hemoglobin ≥10; **2**: <10	gm/dl
P1	Vascular events in dabigatran 110mg subgroup*	**1**: No vascular event happened within the first year of follow-up; **2**: Yes	-
P2	Bleeding (Major and minor bleeding) in dabigatran 150mg subgroup*	**1**: No bleeding event happened within the first year of follow-up; **2**: Yes	-

* BMI: body mass index, GFR: glomerular filtration rate, TIA: transient ischemic attack, NSAID: non-steroidal anti-inflammatory drug, COX: cyclooxygenase, MI: myocardial infarction, DM: diabetes mellitus CHF: congestive heart failure.

* Liver function abnormality defined as medical history of cirrhosis or abnormal biochemical data when the patients were enrolled (Bilirubin more than 2 times the upper limit of normal, plus 1 or more of aspartate transaminase, alanine transaminase, or alkaline phosphatase more than 3 times the upper limit of normal).

* Vascular events defined as Stroke, systemic embolism and vascular death.

* Major bleeding was defined as a drop in hemoglobin of ≥2 gm/dl (1.2 mmol/l), transfusion of ≥2 packed cells, or symptomatic bleeding in a critical area or organ. Critical areas were intraocular, intracranial (including hemorrhagic stroke), intraspinal, intramuscular with compartment syndrome, retroperitoneal, intra-articular, or pericardial. All other bleeding was considered minor bleeding.
